# Research on a Method for Measuring the Pile Height of Materials in Agricultural Product Transport Vehicles Based on Binocular Vision

**DOI:** 10.3390/s24227204

**Published:** 2024-11-11

**Authors:** Wang Qian, Pengyong Wang, Hongjie Wang, Shuqin Wu, Yang Hao, Xiaoou Zhang, Xinyu Wang, Wenyan Sun, Haijie Guo, Xin Guo

**Affiliations:** 1 College of Mechanical and Electrical Engineering, Inner Mongolia Agricultural University, Hohhot 010020, China; wpengyong@163.com (P.W.); a1124279970@163.com (X.W.); 15287421657@163.com (W.S.); imaunews@126.com (X.G.); 2 Inner Mongolia Engineering Research Center of Intelligent Equipment for the Entire Process of Forage and Feed Production, Hohhot 010020, China; 3 Chifeng Xinda Machinery Manufacturing Co., Ltd., Chifeng 025556, China; 15004761608@126.com (H.W.); haoyang0913@163.com (Y.H.); 4 Agricultural and Animal Husbandry Technology Extension Center of Inner Mongolia Autonomous Region, Hohhot 010010, China; 13847149977@139.com (S.W.); 13704715746@163.com (X.Z.); 15771371797@163.com (H.G.)

**Keywords:** agricultural material, pile height measurement, master-slave collaborative operations, binocular stereo vision, stereo matching, linear regression

## Abstract

The advancement of unloading technology in combine harvesting is crucial for the intelligent development of agricultural machinery. Accurately measuring material pile height in transport vehicles is essential, as uneven accumulation can lead to spillage and voids, reducing loading efficiency. Relying solely on manual observation for measuring stack height can decrease harvesting efficiency and pose safety risks due to driver distraction. This research applies binocular vision to agricultural harvesting, proposing a novel method that uses a stereo matching algorithm to measure material pile height during harvesting. By comparing distance measurements taken in both empty and loaded states, the method determines stack height. A linear regression model processes the stack height data, enhancing measurement accuracy. A binocular vision system was established, applying Zhang’s calibration method on the MATLAB (R2019a) platform to correct camera parameters, achieving a calibration error of 0.15 pixels. The study implemented block matching (BM) and semi-global block matching (SGBM) algorithms using the OpenCV (4.8.1) library on the PyCharm (2020.3.5) platform for stereo matching, generating disparity, and pseudo-color maps. Three-dimensional coordinates of key points on the piled material were calculated to measure distances from the vehicle container bottom and material surface to the binocular camera, allowing for the calculation of material pile height. Furthermore, a linear regression model was applied to correct the data, enhancing the accuracy of the measured pile height. The results indicate that by employing binocular stereo vision and stereo matching algorithms, followed by linear regression, this method can accurately calculate material pile height. The average relative error for the BM algorithm was 3.70%, and for the SGBM algorithm, it was 3.35%, both within the acceptable precision range. While the SGBM algorithm was, on average, 46 ms slower than the BM algorithm, both maintained errors under 7% and computation times under 100 ms, meeting the real-time measurement requirements for combine harvesting. In practical operations, this method can effectively measure material pile height in transport vehicles. The choice of matching algorithm should consider container size, material properties, and the balance between measurement time, accuracy, and disparity map completeness. This approach aids in manual adjustment of machinery posture and provides data support for future autonomous master-slave collaborative operations in combine harvesting.

## 1. Introduction

Joint harvesting of agricultural products represents a critical trajectory in the advancement of agricultural machinery intelligence, playing a vital role in ensuring harvest safety and enhancing efficiency [1,2,3,4]. However, research on the automatic measurement of material pile height in transport vehicle containers within China remains at a nascent stage. Typically, operators rely on visual inspection of the throwing device and the material accumulation in the container or depend on video surveillance combined with experiential judgment. This approach not only significantly hampers harvest efficiency but also considerably increases the labor intensity of the drivers.

With the advancement of intelligent control technology, follow-up unloading techniques during joint harvesting have emerged as a primary development trend [5]. The automatic measurement of material pile height in transport vehicle containers provides reliable informational support for the coordinated operation of harvesters and transport vehicles, as well as for automatic following technology. The essential technologies required to achieve automatic unloading functionality in joint harvesting include dynamic detection of trailer hoppers, automatic planning of drop points, prediction of material pile states, and control of the unloading arm movement [6]. These four technologies are interdependent and closely integrated, forming a closed-loop control system. Among them, the detection of the trailer hopper is fundamental to achieving automatic unloading of crops, while accurate prediction of the material drop position and pile state is crucial for enhancing unloading efficiency and optimizing the utilization of hopper space [7]. Liu et al. [8] developed a computational model to simulate the accumulation process of granular materials. This model predicts particle distribution, spillage volume, and spillage location based on the geometry of the trailer, the impact location, and the direction of particle flow. Dai et al. [9] conducted physical model experiments and numerical simulations to test the influence of fabric orientation on the angle of repose of materials, discovering that the orientation angle of the deposition plane minimizes between 30° and 45°. Wang et al. [10] proposed a dynamic uniform unloading method based on three-dimensional point cloud data, which addresses the issue of uneven unloading in trailer hoppers during mine transport by real-time adjustment of the unloading point.

With the advancement of machine vision technology, vision sensors and LiDAR sensors installed on agricultural robots or mobile platforms have been widely utilized for field operations [11,12,13,14,15]. Li et al. [16] proposed a lightweight, improved YOLOv5s model for detecting pitaya fruits under both daytime and nighttime lighting conditions, successfully deploying it on an Android device. Evarist et al. [17] developed a model for detecting pesticide residues in the edible parts of vegetables, including tomatoes, cabbages, carrots, and green peppers. Jahari et al. [18] developed a machine vision system that evaluates the quality of rice during harvest using dual light assessment, employing low-cost webcams to identify unwanted materials and damaged grains. Similarly, Jiang et al. [19] developed a grain unloading system integrating radar and camera technologies, proposing a method for recognizing the state of grain trucks. By evaluating various LiDAR sensors, they established a data processing framework to enhance the accuracy of grain truck contour estimation across different environments. In another study [20], a monocular vision system was proposed to guide tractors. This system captures images as the vehicle traverses crop rows, identifying heading and offset errors to correct the steering angle. Ball et al. [21] described a vision-based obstacle detection and navigation system that combines global positioning system (GPS) and inertial navigation system (INS) for obstacle detection and visual guidance. Santos et al. [22] proposed a real-time obstacle avoidance method for steep farmland based on local observations, which was successfully tested in vineyards. They plan to integrate LiDAR with depth cameras in the future to improve elevation mapping perception.

However, research on dynamic detection of loading status in grain transport vehicles using machine vision is relatively limited, primarily focusing on the automatic filling of silage between self-propelled forage harvesters and trailers [23]. The reliability of the harvester and grain transport vehicle coordination system largely depends on the detection accuracy of the trailer hopper [24]. Chen Ying et al. [25] used CCD cameras to capture side views of piles from multiple angles and calculated the volume of large piles by measuring the area of each cross-section. Liu et al. [26] applied a machine vision-based method to detect the loading status using the contact line between the grain and the bin, dividing the grain bin into four regions and setting thresholds to determine their loading status. Kyoto University developed a combine harvester robot [27] that utilizes a laser scanner to position the combine harvester’s auger at the center of the grain container for unloading. Zhang et al. [28] achieved clear and complete extraction of grain bin edges, enabling real-time dynamic monitoring of the grain loading status. This approach prevents grain spillage, enhances the efficiency of master-slave collaborative operations, and improves the monitoring capabilities between the harvester and the grain transport vehicle. Cui et al. [29] proposed an automated grain unloading method based on stereo vision and geometric modeling. This method utilizes nonlinear least squares fitting to map the collected grain pile height data to unloading time, averaging the results from multiple datasets. Liu et al. [30] developed and validated an automated unloading system for grain that, without the need for markers or manual vehicle position coordination, monitors truck fullness using a stereo camera-based perception system to estimate the grain height relative to the truck’s edge.

In the previously mentioned study, due to the insufficient dimensionality of monocular vision data, the complexity of the field environment, varying lighting conditions, and the diverse types of trailers, the accuracy of traditional image detection methods for material transport vehicle bins fails to meet the requirements for cooperative harvesting between harvesters and grain transport vehicles. Although LiDAR can be used to obtain 3D data, its high cost results in limited related research. In studies related to stereo vision, the focus has been primarily on the accumulation state and 3D point clouds, or on calculating material accumulation height over time. However, the real-time and accurate measurement of material accumulation height is not feasible due to the varying types of transport vehicles and the influence of environmental factors such as vehicle speed and wind speed.

To obtain the material accumulation height in transport vehicle bins, this paper proposes a method for measuring the material accumulation height based on stereo vision. This method calculates the disparity value for each pixel by comparing the pixel differences between the left and right images captured by the stereo camera. Subsequently, using the disparity values and camera parameters, a disparity map is generated, which enables the calculation of the distances from the bin bottom and the surface of the accumulated material to the stereo camera. The difference in distance measurements between the empty and loaded states is then used to determine the material accumulation height. Finally, linear regression is applied to the accumulation height results to obtain the corrected material accumulation height. This method not only reduces manual labor but also provides reliable information support for the cooperative operation of harvesters and transport vehicles, as well as for autonomous following technology.

## 2. Materials and Methods

### 2.1. Binocular Distance Measurement Method

**Figure 1:**
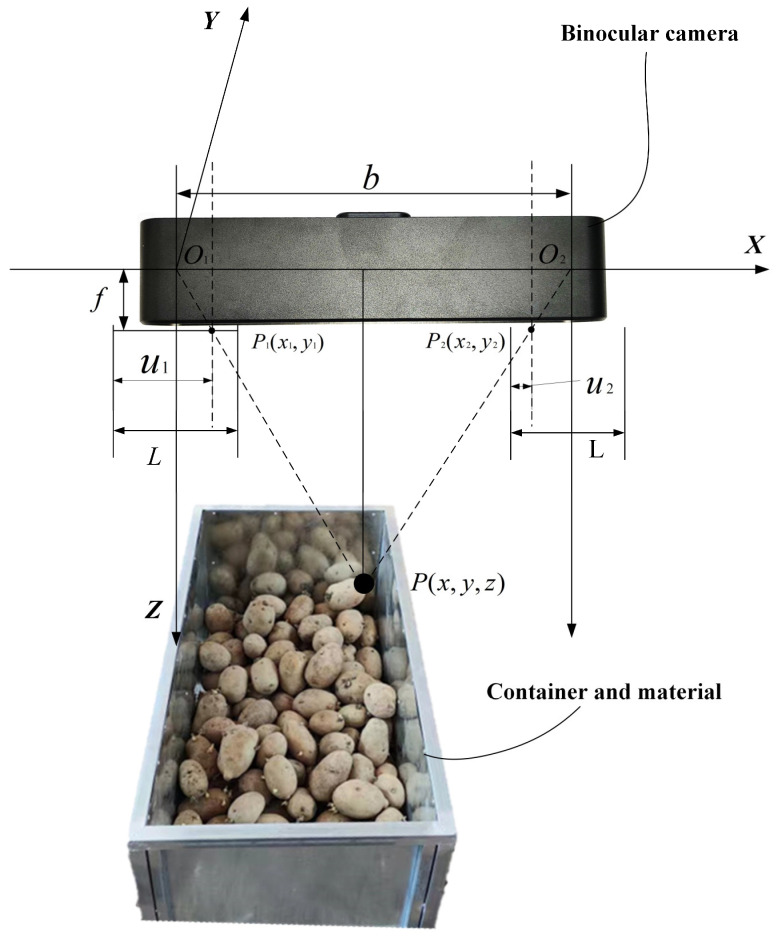
Principle of triangulation.

When imaging on a photosensitive element, the spatial depth information of an object is lost. Compared to monocular vision, binocular vision operates similarly to human vision by leveraging the parallax between camera positions and utilizing the disparity in pixel coordinates between the left and right images. Through triangulation, the spatial coordinates of feature points are calculated, allowing for the retrieval of depth information of the measured target. This process enables more accurate positioning and measurement of objects. The experiment was conducted in a soil trough laboratory, where the measured object was a simulated truck bed loaded with potatoes. The experimental conditions combined natural and artificial lighting to create a more realistic environment. The material examined consisted of yellow-skinned potatoes grown in Inner Mongolia, characterized by a smooth surface and regular shapes, predominantly circular or oval. The simulated truck was made of steel, featuring a white-gray color. The principle of triangulation is illustrated in Figure 1.

Let $XYO_{1}Z$ be the world coordinate system, coinciding with the left camera coordinate system, with $O_{1}$ as the optical center of the left camera and $O_{2}$ as the optical center of the right camera. The X-axis is oriented horizontally to the right and is labeled as the lateral axis. It is parallel to the optical axis of the camera and represents the horizontal positional changes of objects. The Y-axis is perpendicular to the X-axis, and the plane formed by the X and Y axes coincides with the real imaging plane, which, in this experiment, is parallel to the horizontal plane. The Z-axis extends along the depth direction of the image, perpendicular to the XY plane, and represents changes in the depth of objects. The focal length of both cameras is denoted as f, and the distance b between the optical centers of the left and right cameras represents the baseline of the stereo camera system. Let the binocular camera spatial coordinates of point P be represented as (x,y,z). Since the raw image captured by the camera is inverted, the camera applies a flipping process to produce an upright image. In Figure 1, $P_{1}$ and $P_{2}$ are symmetrical points in the real imaging plane. When the stereo camera captures point P, the images of point P on the left and right camera planes are denoted as $P_{1}(x_{1},y_{1})$ and $P_{2}(x_{2},y_{2})$, respectively. Let L be the length of the imaging planes for both cameras. The distances from the imaging points to the left boundary of the imaging plane, $u_{1}$ and $u_{2}$, are defined as the distances from the imaging points on the left and right cameras, respectively. The $d=u_{1}-u_{2}$ is defined as disparity. According to the principle of similar triangles, it can be determined that:

(1) $$\left\{\begin{array}{l} \frac{x_{2}-x_{1}}{b}=\frac{z-f}{z}\\ u_{1}-u_{2}=d\\ u_{1}=\frac{L}{2}+x1\\ u_{2}=\frac{L}{2}-(b-x_{2}) \end{array}\right.$$

By converting the triangular equation, the desired distance z can be obtained:

(2) $$z=b\frac{f}{d}$$

### 2.2. Binocular Calibration

The binocular camera used in the experiment is the MK4231 model produced by Xintong Technology. The detailed parameters are listed in Table 1.

**Figure 2:**
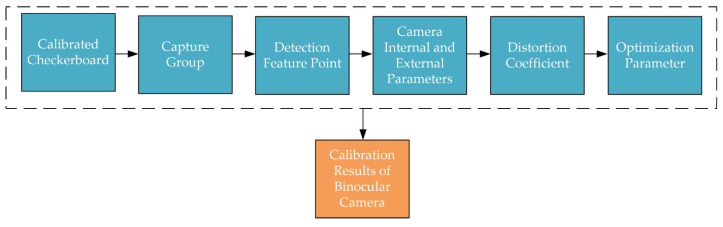
Zhang’ calibration steps.

To determine the relative positional relationship between the image coordinate system and the world coordinate system, the binocular camera requires parameter calibration to obtain the camera’s intrinsic and extrinsic parameters. The main camera calibration methods include the traditional calibration method, self-calibration method, active vision calibration method, and Zhang’s calibration method [31,32,33]. Among these, Zhang’s calibration method first solves for the optimal solution of the camera’s intrinsic and extrinsic parameters using a linear model, then employs the maximum likelihood estimation for nonlinear optimization to obtain the camera parameters. This method is simple to implement and highly accurate. Therefore, in this paper, Zhang’s calibration method is employed, using MATLAB (R2019a)’s built-in stereo camera calibrator APP for the calibration of the binocular camera. The calibration steps are illustrated in Figure 2.

**Figure 3:**
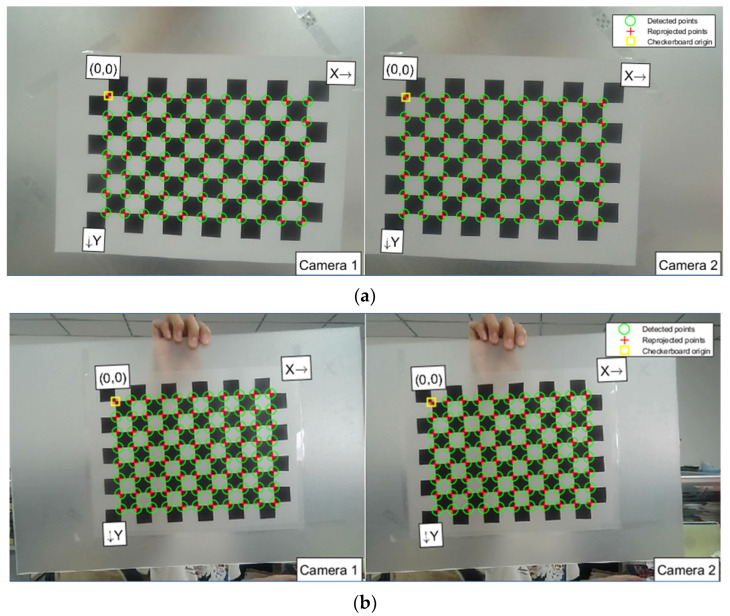
The corner extraction results of the checkerboard. (**a**) Calibration paper; (**b**) Calibration plate.

First, the left and right images of a checkerboard calibration board are captured using a stereo camera. The camera and the board are positioned to ensure the entire calibration board is visible in both images. By adjusting the relative position and angle, the checkerboard appears in different locations across the images. In this experiment, 15 usable sets are gathered for each calibration material. To achieve optimal calibration parameters and minimize distortion, both calibration paper and the board are used in the material pile height measurement experiment. The checkerboard images, along with the dimensions of the black and white squares, are input into the calibration program, which automatically extracts and marks the corners of the checkerboard. The corner extraction results for the two materials are displayed in Figure 3.

In Figure 3, the green circles represent the detected corners, the red crosses indicate the reprojected points, and the yellow squares denote the origin of the checkerboard. For precise corner measurement during the extraction process, the calibration checkerboard should occupy 50% to 80% of the entire image. In this experiment, it occupied approximately two-thirds of the image, leaving space at the edges and corners to avoid severely distorted areas that could affect calibration results. This careful approach ensures the accuracy of the material pile height measurement.

**Figure 4:**
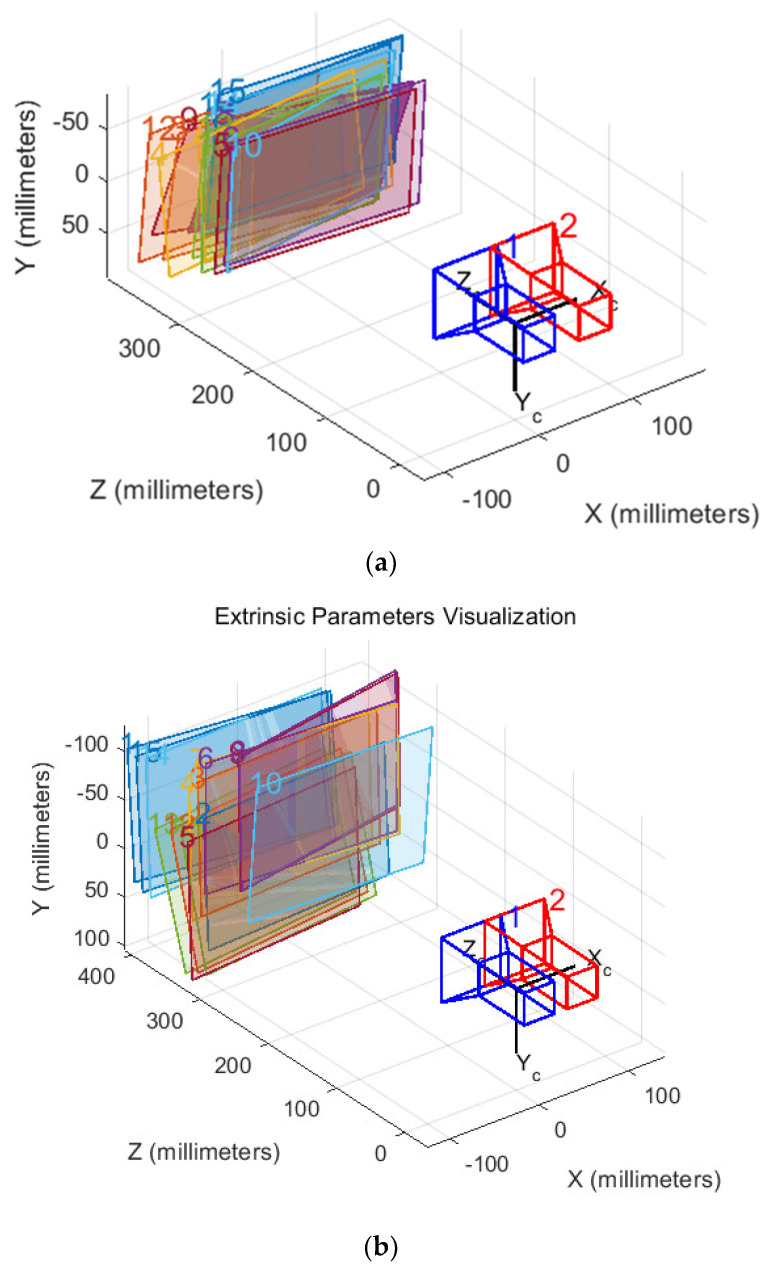
The relative position between the binocular camera and the calibration board. (**a**) Calibration paper; (**b**) calibration plate.

Next, the stereo camera is calibrated. The program utilizes the extracted corners and the actual dimensions of the checkerboard squares to determine the coordinates of the corners in both the world and image coordinate systems. This information is essential for calculating the intrinsic and extrinsic parameter matrices of the left and right cameras, which are crucial for accurate depth perception. The initial parameters are then used to estimate the distortion parameters of both cameras, followed by further optimization. Figure 4 illustrates the relative position of the stereo camera and the checkerboard during calibration.

**Figure 5:**
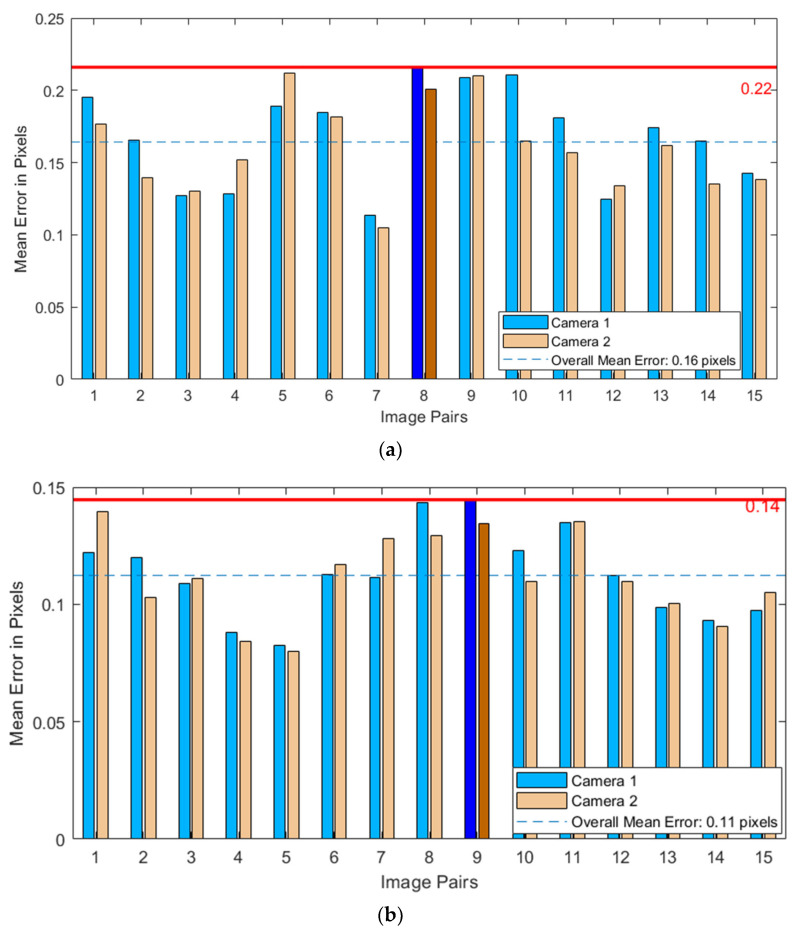
The reprojection errors of the chessboard calibration. (**a**) Calibration paper; (**b**) calibration plate.

After performing calibration using both calibration paper and calibration board, the accuracy of the calibration is assessed by the reprojection error. The smaller the average reprojection error, the more precise the calibration results. Figure 5 illustrates the reprojection errors for chessboard calibrations using materials of two different types.

Comparing the calibration results of chessboards made from two different materials, the average reprojection errors for calibration paper and calibration board were 0.16 pixels and 0.11 pixels, respectively, indicating that the calibration board yields more accurate results. This superior performance can be attributed to the board’s higher manufacturing precision, flatness, and rigidity. In contrast, paper may develop wrinkles or bends due to improper placement, adversely affecting calibration accuracy. Therefore, a calibration board was employed in this experiment, leading to the post-analysis of various parameters of the binocular system and the construction of the imaging model for the stereo camera.

The stereo camera parameters obtained through Zhang’s calibration method are presented in Table 2. These parameters include the camera’s intrinsic matrix, rotation matrix, distortion coefficients, and translation matrix.

### 2.3. Polar Correction

The relative positioning of stereo cameras is crucial for accurately converting disparity information into depth information. In an ideal stereo vision system, the two cameras are aligned on the same horizontal plane, ensuring that the captured images are perfectly aligned in the vertical direction. This setup allows for the calculation of disparity solely along the horizontal axis. However, achieving perfect horizontal alignment is often challenging due to factors such as human error. Consequently, the cameras may not be perfectly level, resulting in images captured at different vertical planes and introducing vertical disparity. This vertical disparity complicates disparity calculation and depth estimation, as it requires consideration of both horizontal and vertical deviations.

Upon calibration, the intrinsic and extrinsic parameters obtained can be used to rectify the raw images from the stereo cameras. This rectification process aligns the images on the same horizontal plane, simplifying disparity calculation from a two-dimensional to a one-dimensional problem. This reduction in complexity decreases computational load and minimizes the likelihood of errors in stereo matching.

**Figure 6:**
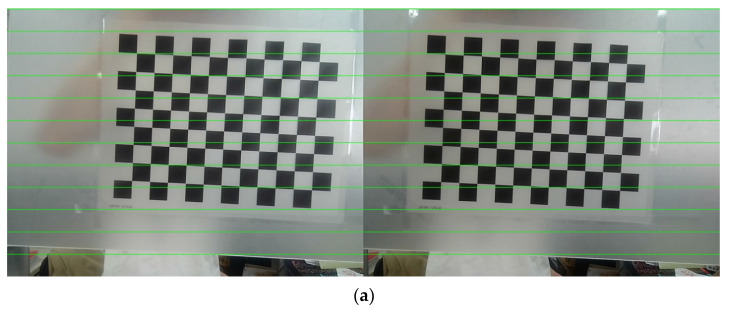

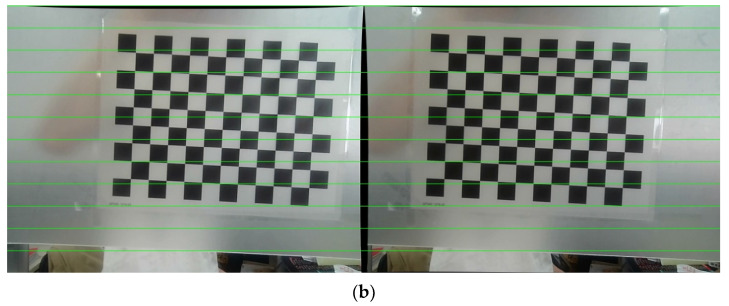
Polar correction. (**a**) Before correction; (**b**) after correction.

While stereo matching can technically be performed without polar rectification, this method involves an exhaustive search across all pixels in both the left and right images to find corresponding points. Such an exhaustive search significantly reduces efficiency and is particularly prone to errors in low-texture regions. Therefore, applying polar geometric constraints to align the polar lines with the imaging plane’s horizontal axis is essential. This alignment simplifies the matching process by reducing it to a one-dimensional search along the horizontal direction, enhancing both computational efficiency and accuracy. As illustrated in Figure 6, after polar rectification, the search for corresponding points is constrained to the polar line on which the target pixel resides. This restriction narrows the search space from the entire image to a single polar line, thereby reducing the likelihood of matching errors and improving matching speed.

Through comparison, it is observed that before polar rectification, the positions of feature points, such as the corners of a chessboard, in the calibration images are not horizontally aligned. However, after applying polar rectification using the intrinsic and extrinsic parameters obtained from calibration, the chessboard images in the left and right images become horizontally aligned. The polar lines in the rectified images are parallel, and the vertical coordinates of corresponding points are consistent. This alignment ensures that corresponding pixels are located at the same vertical position, thereby simplifying and enhancing the efficiency of matching corresponding points in subsequent experimental processes.

### 2.4. Stereo Matching Algorithms

Stereo matching is a critical step in binocular stereo vision, aimed at identifying corresponding pixels in the left and right images to compute disparity and map 2D image planes to 3D depth information. Disparity refers to the horizontal displacement difference of a pixel between the two images; a larger disparity value indicates a closer object, while a smaller value suggests greater distance. By utilizing the disparity and known intrinsic and extrinsic parameters of the stereo cameras, depth information and disparity maps can be calculated to determine the object’s distance from the camera. The quality of these disparity results directly impacts measurement accuracy. Stereo matching methods are categorized into three main types based on their optimization theories: block matching (BM), global correspondence (GC), and semi-global block matching (SGBM). Due to the complexity and lengthy processing times of global stereo matching algorithms, they have been removed from OpenCV. Although recent advancements in deep learning have improved stereo matching accuracy, discrepancies in image style between training and test datasets, along with a lack of parallax labels, can hinder performance. In agriculture, particularly during the harvest of various crops, deep learning-based 3D matching algorithms may face challenges in these contexts. Consequently, this study focuses on analyzing and comparing BM and SGBM. The primary objective is to determine the optimal disparity value d that minimizes the pixel differences between the left and right images within a specified local window or path. This approach aims to enhance the accuracy of stereo vision by optimizing image matching.

In BM, disparity estimation is typically performed within a small window. This localized approach allows for more precise calculations of pixel disparities, enhancing the overall accuracy of depth perception, the principle is as follows Equation (3). Let $d\left(x,y\right)$ denote the disparity value at the point $\left(x,y\right)$. The notation $arg{min}_{d}$ refers to the value of d that minimizes the subsequent expression. The coordinates $\left(x^{\prime },y^{\prime }\right)$ represent the pixel points within the local window $W\left(x,y\right)$, which is centered at $\left(x,y\right)$. $I_{L}\left(x^{\prime },y^{\prime }\right)$ denotes the pixel intensity of the left image at the point $\left(x^{\prime },y^{\prime }\right)$, while $I_{R}\left(x^{\prime }+d,y^{\prime }\right)$ indicates the pixel intensity of the right image at the point $\left(x^{\prime }+d,y^{\prime }\right)$. Here, d signifies the disparity value, representing the horizontal distance between matching points in the left and right images.

(3) $$d\left(x,y\right)={argmin}_{d}\sum_{\left(x^{\prime },y^{\prime }\right)\in W\left(x,y\right)}{\left(I_{L}\left(x^{\prime },y^{\prime }\right)-I_{R}\left(x^{\prime }+d,y^{\prime }\right)\right)}^{2}$$

The core idea of SGBM algorithm is to consider the global information in the image, not just the local neighborhood information, the principle is as follows Equation (4). Let k denote the radius of the search window. The term $\omega_{ij}$ represents the weight coefficient, while $C\left(x+i,y+j,d\right)$ denotes the cost function used to compute the matching cost between the left and right images at disparity d for the point $\left(x+i,y+j\right)$. This cost is typically based on the squared differences in pixel intensities.

(4) $$d\left(x,y\right)=arg\;{min}_{d}\left(\sum_{i=-k}^{k}\sum_{j=-k}^{k}\omega_{ij}\cdot C\left(x+i,y+j,d\right)\right)$$

**Figure 7:**
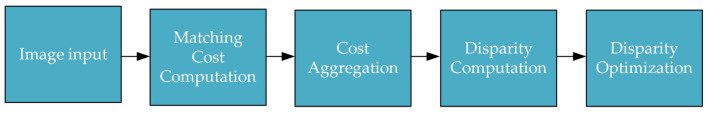
Basic workflow of stereo matching.

Figure 7 illustrates the basic process of stereo matching.

The block matching (BM) [34] algorithm is a local matching method that relies on window intensity values, determining matches based on the window with the highest similarity. While BM is simple and computationally efficient, it often produces mismatches in areas with sparse or repetitive textures. In contrast, the semi-global block matching (SGBM) [35] algorithm combines local and global matching strategies by aggregating costs from multiple directions, enhancing matching accuracy, and effectively addressing challenges in weakly textured and edge regions. Although SGBM has higher computational complexity, it is well suited for applications that require high precision.

In practical applications of stereo vision for measuring the pile height of agricultural products on transport vehicles, it is essential to select an appropriate stereo matching algorithm based on the type of agricultural product and its physical characteristics. This study utilizes the OpenCV library to perform stereo matching on preprocessed images of the experimental material box loading states using both the BM and SGBM algorithms. The performance and efficiency of these two algorithms are then analyzed in the context of this experiment.

### 2.5. Method for Measuring the Height of Piled Materials

**Figure 8:**
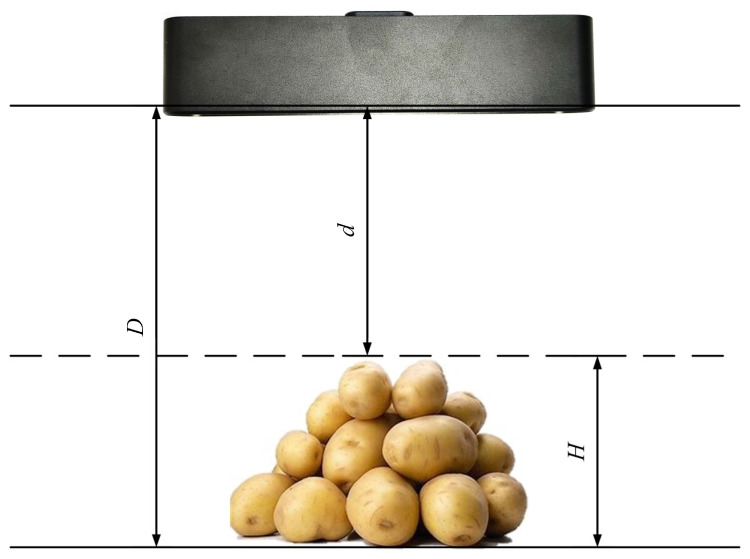
Method for measuring the height of piled materials.

As illustrated in Figure 8, the diagram depicts the method for measuring the height of piled materials on agricultural transport vehicles. By measuring the distance *D* from the bottom of the empty cargo box to the stereo camera and the distance d from the surface of the piled potatoes to the stereo camera during the current loading trial, the pile height H at various positions during loading can be calculated using Equation (5). The resulting measurements provide loading information to assist the driver in making subsequent decisions.

(5) H=D−d

**Figure 9:**
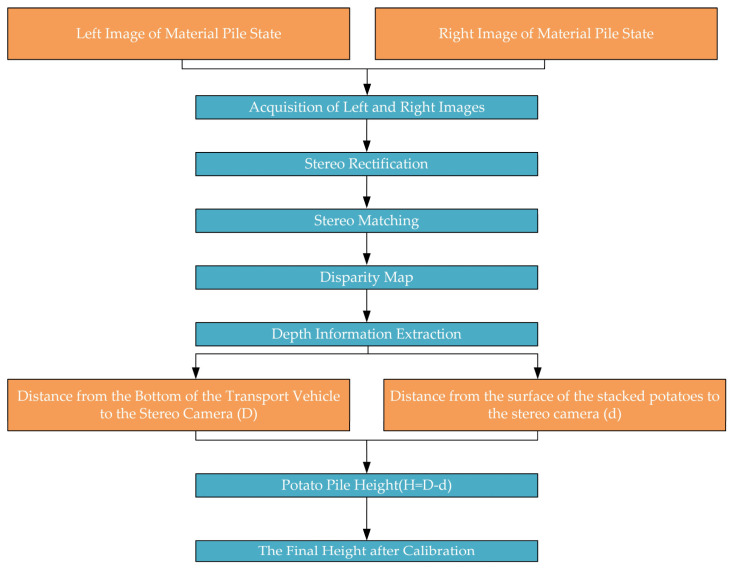
The process of measuring the piled height of potatoes.

By performing stereo matching on the rectified left and right images, a disparity map can be generated, thereby obtaining depth information. This allows for the calculation of the distances from the bottom of the material box and the surface of the piled materials to the stereo camera. The differences between the distances measured during the empty and loaded states are then used to determine the pile height of the materials. Finally, a linear regression is applied to the calculated pile heights to derive the corrected material pile height. The overall workflow is illustrated in Figure 9.

## 3. Results and Analysis

### 3.1. Disparity Map Acquisition Effect

**Figure 10:**
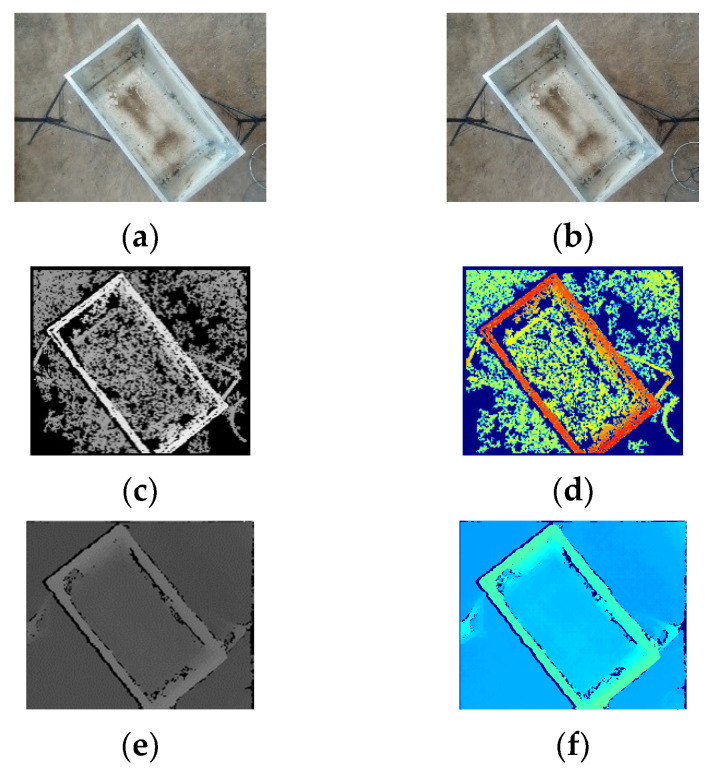
The image under no-load conditions. (**a**) Left image; (**b**) right image; (**c**) BM disparity map; (**d**) BM pseudo-color map; (**e**) SGBM disparity map; (**f**) SGBM pseudo-color map.

**Figure 11:**
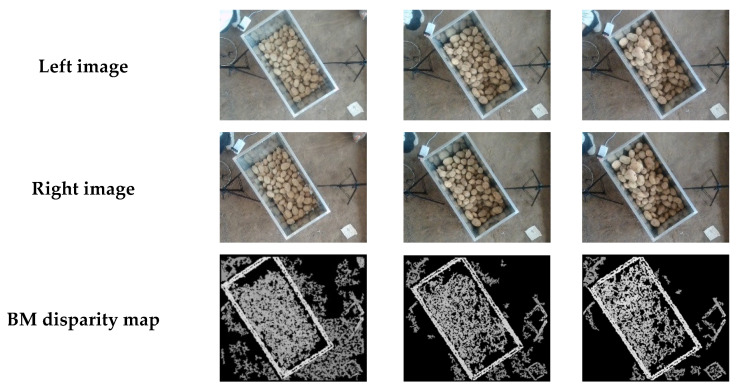

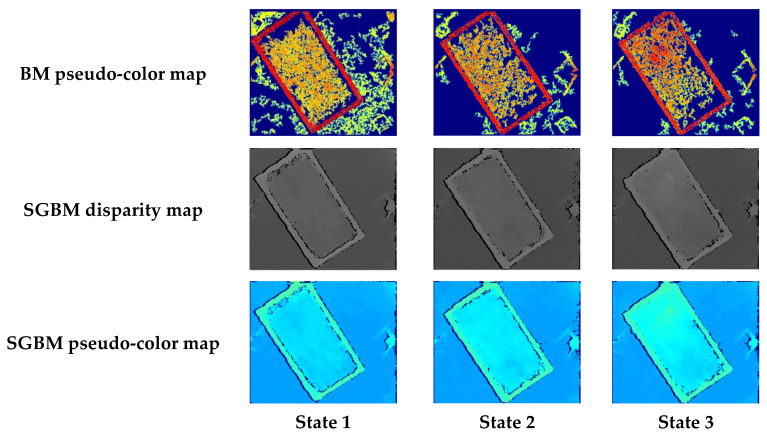
Images of three different load conditions.

In this experiment, the materials used were potatoes grown in the Inner Mongolia region. Initially, the distance D between the bottom of the container and the surface of the piled materials to the stereo camera was measured. The experimental design included the empty state of the container and three different piling states of potatoes within the container. By moving the container, the distance d between the potato surface and the stereo camera at ten different positions was measured. Figure 10 and Figure 11, respectively, show the raw images, disparity maps, and pseudo-color maps obtained from the left and right cameras processed by two stereo matching algorithms under the empty container state and three loading conditions. The pseudo-color maps significantly enhance the visual effects and data readability of the disparity maps and other grayscale images through color mapping techniques.

From Figure 10 and Figure 11, it can be observed that both the BM and SGBM matching algorithms can recover disparity information from stereo images to a certain extent. The disparity map produced by the BM algorithm has numerous holes, but the overall features of the main contour of the material box and the height information of the material accumulation inside the box are relatively clear. However, the ground shows many holes with no depth information. In contrast, the disparity map generated by the SGBM algorithm has fewer holes, is less affected by lighting conditions, and the ground does not exhibit large areas of missing depth information. However, a black boundary appears at the connection between the edge of the material box and the ground.

### 3.2. Distance Measurement Results

After obtaining the disparity maps for the left and right images, distance information for each pixel can be recovered based on the principles of stereo vision. This allows for the determination of the distance from each pixel to the stereo camera, ultimately providing the distance from the object being measured to the stereo camera.

Firstly, the distance between the bottom of the empty cargo box and the surface of the piled potatoes to the stereo camera is measured. The height of the camera relative to the cargo box and the ground remains unchanged. By moving the cargo box forwards and backwards, distances at various positions within the box are measured. A program is developed to allow for the selection of pixel points on the disparity map, calculate the corresponding distance information for these points, and output the results. To validate the accuracy of distance measurements, the experiment compares the BM and SGBM algorithms.

For the empty cargo container, the distance from the stereo camera to the bottom of the container was measured ten times using two different algorithms. The measured values were compared with the ground truth, and both the measurements and their relative errors are shown in Table 3.

Utilizing the capability of stereo cameras to perform multiple distance measurements of the target object within a short time frame, this experiment calculated the average values from ten measurements obtained using two algorithms. The average values were then compared with the ground truth to derive the mean absolute error (MAE) and, subsequently, the mean relative error (MRE). The average measurement MAE and MRE for the BM and SGBM algorithms under unloaded conditions are presented in Table 4.

Based on Table 4 and Table 5, it is observed that for multiple measurements at 1360 mm, the average relative errors for the BM and SGBM algorithms are 4.90% and 3.47%, respectively. This indicates the stability and reliability of these two algorithms in distance measurement for this experiment. Additionally, the maximum relative error is less than 6%, further demonstrating the high precision of both algorithms.

**Figure 12:**
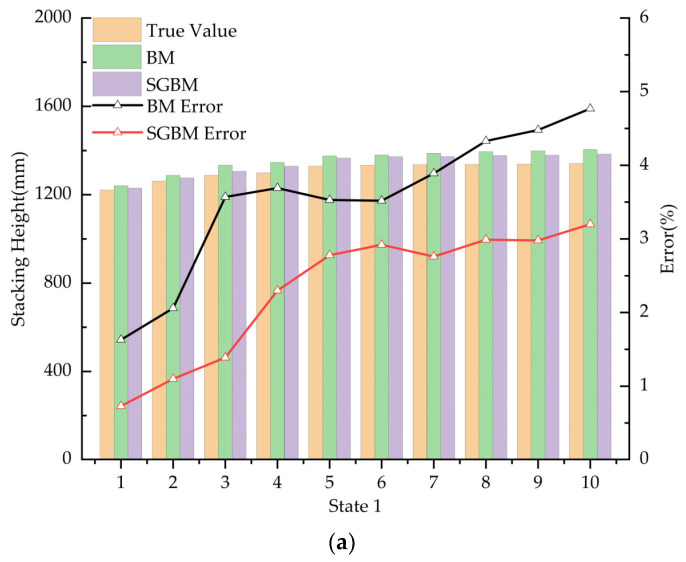

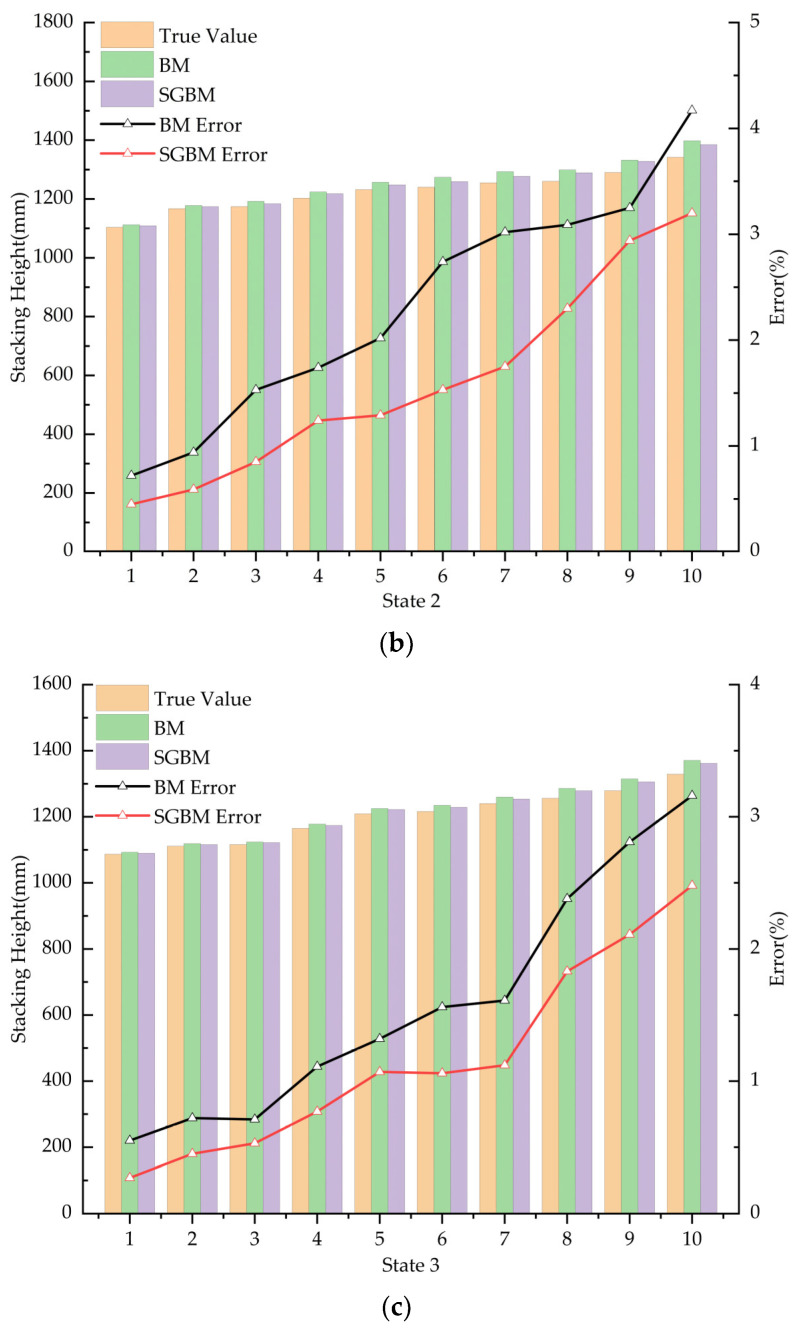
The distance measurement results between the surface of stacked potatoes and the stereo camera under three different conditions. (**a**) State 1; (**b**) state 2; (**c**) state 3.

Subsequently, the distance measurements between the surface of potatoes and the stereo camera at ten different locations under three distinct stacking conditions are illustrated in Figure 12.

As illustrated in Figure 12, the distance measurement error of the stereo camera is relatively small at close range; however, as the distance increases, the error progressively enlarges and accuracy diminishes. This phenomenon occurs because, at close distances, the camera captures a higher number of effective pixels, resulting in a clearer disparity map and thus higher measurement precision. Conversely, at greater distances, the camera’s field of view becomes broader, making the image more complex. This complexity leads to less accurate disparity maps, increasing the error in distance information, reducing the number of effective pixels, and ultimately resulting in decreased measurement accuracy.

To further assess the reliability and timeliness of the two different matching algorithms employed in this study, the measurement times for each algorithm were computed. The final results are presented in Table 5.

In this experiment, within a measurement distance range of 1360 mm, the SGBM algorithm demonstrated superior accuracy compared to the BM algorithm, achieving an average precision improvement of 1.43%. However, the SGBM algorithm had an average computation time that was 46 ms longer than that of the BM algorithm. Both algorithms maintained errors below 7% and processing times under 100 ms, thus satisfying real-time distance measurement requirements. The BM algorithm offers faster image processing and higher computational efficiency, making it more suitable for applications requiring rapid image analysis. In contrast, the SGBM algorithm produces disparity maps with fewer environmental noise points and voids, resulting in clearer and more precise maps with lower measurement errors. Therefore, it is better suited for applications where high measurement accuracy is critical. While both algorithms meet the experimental requirements, the choice between them should be based on specific field conditions during actual harvesting, taking into account external factors such as speed and accuracy needs.

### 3.3. Results of Material Pile Height Measurement

In this experiment, the average distance D between the bottom of the cargo compartment and the stereo camera was first measured under unloaded conditions, and the average distance d between the potato pile surface and the stereo camera was measured under loaded conditions. Subsequently, the potato pile height H at various positions was computed using Equation (3).

In statistical analysis, function results inherently contain errors due to variable uncertainties, a phenomenon known as error propagation. This issue also affects the calculation of material pile heights in this experiment. Figure 12 illustrates that there exists an approximately linear relationship between the true values and the measured values. To enhance measurement accuracy, a linear regression analysis was performed on the measured potato pile heights H in comparison to the true values, resulting in a calibration model. This laptop runs on Intel(R) Core(TM) i5-8250U, GPU NVIDIA GeForce MX110, RAM 8 G, Windows 10 (64-bit), program development software PyCharm 2023, and the algorithm is written in Python (3.8). The 30 datasets were organized in ascending order, from which 15 datasets were uniformly selected for fitting the regression equation. The remaining 15 datasets were utilized to test and evaluate the performance of the calibration model. On the PyCharm platform, a model was constructed using the Python (3.8) scikit-learn library. The training dataset was utilized to determine the regression model coefficients, thereby establishing the linear relationship. Subsequently, the model was evaluated, and predictions were made using the test dataset. Finally, scatter plots were generated using matplotlib to visually compare the true values with the predicted values from the linear regression model, facilitating an intuitive understanding of the model’s performance. The experiment was conducted under three different conditions, resulting in a total of 30 datasets. The minimum distance measurement recorded was 1087 mm, while the maximum distance measured was 1342 mm. These datasets included the real and measured distances between the stereo camera and the potato pile surface, as well as between the stereo camera and the bottom of the cargo compartment. Using Equation (3), 30 sets of potato pile height data were computed.

**Figure 13:**
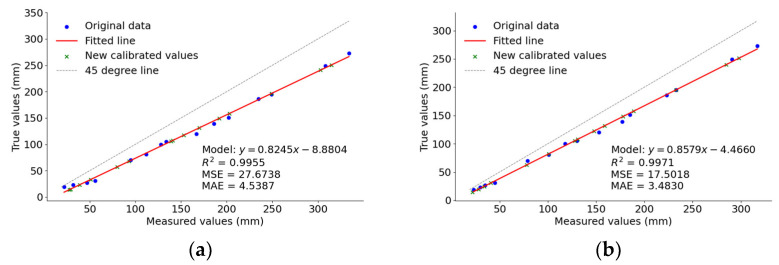

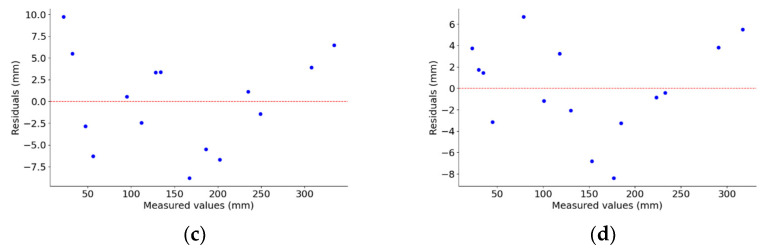
Regression model and evaluation metrics. (**a**) BM measurement values and calibrated values; (**b**) SGBM measurement values and calibrated values; (**c**) residual plot of the BM regression model; (**d**) residual plot of the SGBM regression model.

In order to evaluate the validity of the linear regression model, the performance of the calibration model was assessed. The results of this assessment are presented in Figure 13.

As shown in Figure 13a,b, the R^2^ (coefficient of determination) is a statistical measure of model fit, with values closer to 1 indicating better fit. The BM achieved an R^2^ of 0.9955, while SGBM reached 0.9971, both indicating excellent fit. Mean squared error (MSE) quantifies the difference between predicted values and actual observations, with smaller values indicating reduced prediction error. SGBM’s MSE is smaller than that of BM, suggesting that SGBM has a lower prediction error. Mean absolute error (MAE) measures the average absolute difference between predicted and actual values, with smaller values indicating higher prediction accuracy. Similarly, SGBM’s MAE is also lower than that of BM, reinforcing its superior predictive performance. This demonstrates that the SGBM algorithm’s calibration model has better explanatory power, lower post-correction error, and higher accuracy compared to the BM algorithm’s calibration model. As shown in Figure 13c,d, the residuals of both calibration models for the two algorithms are randomly distributed around zero, showing no significant patterns or trends. Additionally, the residuals fall within the range of −10 mm to 10 mm, indicating a good fit between the models and the data.

**Figure 14:**
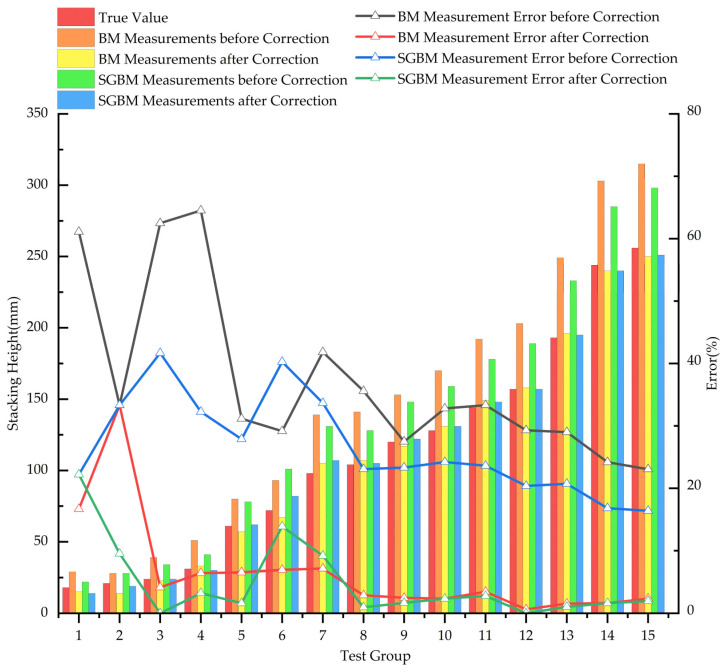
Comparison of pile heights and errors before and after calibration.

To validate the improvement in measurement accuracy provided by the calibration model, the pile height data before and after calibration were compared using the test set data. The comparison results are shown in Figure 14.

As shown in Figure 14, the errors before and after calibration are relatively large at lower stack heights. This is due to the definition of relative error and the nature of the values involved; relative error is the ratio of absolute error to the actual value. When the actual value is small, the denominator is also small, resulting in a larger relative error. Additionally, in this application scenario involving a material transport vehicle, the height of the vehicle’s cargo box is significantly greater than the first three data points presented in Figure 14. To better analyze the calibration effects, data with stack heights below 30 mm were excluded from the comparison of average relative errors.

The results indicate that after calibration, the values obtained through both the BM and SGBM algorithms are much closer to the true values, with significantly reduced relative errors. However, the overall error for the BM algorithm is comparatively higher than that of the SGBM algorithm. The average errors are summarized in Table 6.

As shown in Table 6, after calibration, the mean relative errors for both algorithms are below 4%. Specifically, the BM algorithm’s mean relative error decreased by 29.75% after calibration, while the SGBM algorithm’s mean relative error decreased by 21.86%. The BM algorithm shows a more pronounced reduction in error for smaller values after calibration, whereas the SGBM algorithm is more effective in reducing errors for larger values after calibration. Overall, the measurement results after linear regression calibration are significantly more accurate, substantially reducing measurement errors and thereby enhancing the reliability and precision of the data. This improved accuracy provides more precise input for subsequent algorithms, such as those used in intelligent harvesting, ultimately improving the overall performance of subsequent data processing and analysis.

## 4. Discussion

Currently, the measurement of material pile height in grain transport vehicles during the combine harvesting process primarily relies on manual observation, which is both unsafe and inaccurate. This research applies binocular vision to agricultural harvesting, proposing a method to measure material pile height during the harvest process using a stereo matching algorithm. The method assesses pile height by comparing distance measurements in two states: an empty container and a loaded one. The experiments demonstrate the algorithm’s effectiveness and superior performance in this application. Additionally, a linear regression model is utilized to process the stack height data, which enhances the accuracy of the measurements. This advancement is particularly significant for the automation and intelligent development of agriculture, especially during the combined harvesting process.

Conducted in a soil trough laboratory using potatoes as the stacking material, this study has certain limitations. Different materials exhibit distinct properties, and factors such as ambient lighting may influence measurement results. In practical harvesting operations, the appropriate matching algorithm can be selected based on vehicle box size and material characteristics, balancing measurement time, accuracy, and disparity map voids. To measure stack height, the material bin was manually moved, and multiple readings were taken at the same position to obtain average distance data, while minimizing vibration frequency and amplitude. Future research will investigate the stability and accuracy of this method under various vibration conditions and explore the efficacy of non-traditional algorithms. Furthermore, we aim to examine the integration of binocular cameras with radar technology for measuring material stack height. This approach will enable a comprehensive evaluation of different algorithms in terms of computational efficiency, measurement accuracy, and adaptability to varying environmental conditions.

## 5. Conclusions

This paper proposes a method for measuring the height of material piles inside transport vehicle containers based on binocular vision. The method uses a stereo camera and stereo matching algorithms to measure the distance from the camera to the bottom of the container before loading and to the surface of the piled material during loading. This approach provides real-time measurements of material pile height, effectively addressing the challenges of measuring pile height during the harvesting process and supplying valuable information for subsequent decision making in combine harvesting. The BM and SGBM algorithms utilized in this study are characterized by their strong reliability and short processing times, making them suitable for real-time height calculation and monitoring. Additionally, both algorithms require minimal hardware, which helps reduce implementation costs. Furthermore, a linear regression model is introduced to calibrate the height measurements, mitigating issues related to measurement errors and providing accurate data support for intelligent harvesting. Comparative analysis of the accuracy and processing time of these two algorithms shows that both meet the requirements for precision and real-time performance in combine harvesting. This method, grounded in binocular vision theory, significantly enhances efficiency and safety compared to manual observation during on-site grain unloading. Moreover, it not only provides early warning information for manual adjustments of equipment but also offers data support and innovative solutions for intelligent recognition and pose control in future autonomous collaborative harvesting operations.

## Figures and Tables

**Table 1:** Camera parameters.

Items	Parameters
Resolution	3840 × 1080, synchronized frames, 4 MP
Pixel size	3 µm × 3 µm
Frame rate	60 fps
Baseline	60 mm

**Table 2:** Calibration matrices post-stereo camera calibration.

Parameter	Values	
Left camera intrinsic matrix	$\left[\begin{array}{ccc} 485.357 & 0.649 & 333.596\\ 0 & 486.303 & 269.447\\ 0 & 0 & 1 \end{array}\right]$	
Right camera intrinsic matrix	$\left[\begin{array}{ccc} 482.946 & 0.868 & 330.940\\ 0 & 483.900 & 270.351\\ 0 & 0 & 1 \end{array}\right]$	
Left camera distortion coefficients	$\left[\begin{array}{cccc} 0.0682 & -0.1139 & 0.0013 & 0.0003 \end{array}\right]$	
Right camera distortion coefficients	$\left[\begin{array}{cccc} 0.0673 & -0.0952 & 0.001 & 0.0006 \end{array}\right]$	
Rotation matrix	$\left[\begin{array}{ccc} 0.999959649 & 0.000454789 & -0.008971814\\ -0.000438383 & 0.999998229 & 0.001830443\\ 0.008972631 & -0.001826436 & 0.999958077 \end{array}\right]$	
Translation matrix	$\left[\begin{array}{ccc} -60.1358731 & 0.003172372 & -0.49340667 \end{array}\right]$	

**Table 3:** Results of distance measurement at the bottom of the material bin.

Measurement Number	Actual Distance (mm)	BM (mm)	SGBM (mm)	BM Error (%)	SGBM Error (%)
1	1360	1433	1409	5.36	3.60
2		1424	1404	4.70	3.23
3		1416	1412	4.11	3.82
4		1429	1415	5.07	4.04
5		1427	1408	4.92	3.52
6		1431	1415	5.22	4.04
7		1414	1409	3.97	3.60
8		1435	1398	5.51	2.79
9		1431	1409	5.22	3.60
10		1427	1393	4.92	2.42

**Table 4:** Mean absolute error and mean relative error.

Algorithm	Mean Absolute Error (mm)	Mean Relative Error (%)
BM	66.7	4.90%
SGBM	47.2	3.47%

**Table 5:** Measurement time and mean measurement time.

Algorithm	Measurement Number	Measurement Time (ms)	Mean Measurement Time (ms)
BM	1	44	47
	2	52	
	3	43	
SGBM	1	94	93
	2	92	
	3	93	

**Table 6:** Mean relative error.

Algorithm	Mean Relative Error Before Calibration (%)	Mean Relative Error After Calibration (%)
BM	33.45	3.70
SGBM	25.21	3.35

## Data Availability

The original contributions presented in the study are included in the article, further inquiries can be directed to the corresponding author.

## References

[1] Liquan T., Xue L., Yongsen X., Zhao D. (2021). Design and performance test on segmented-differential threshing and separating unit for head-feed combine harvester. Food Sci. Nutr..

[2] Qing J., Yang L., Xiancun Z., Yadong Y., Jing Z., Bin J., Demei M., Yang Y., Yuan F. (2023). Intelligent Control Knowledge-Based System for Cleaning Device of Rice–Wheat Combine Harvester. Int. J. Pattern Recognit. Artif. Intell..

[3] Wu Z., Chen J., Ma Z., Li Y., Zhu Y. (2024). Development of a lightweight online detection system for impurity content and broken rate in rice for combine harvesters. Comput. Electron. Agric..

[4] Zhou W., Song C., Song K., Wen N., Sun X., Gao P. (2023). Surface Defect Detection System for Carrot Combine Harvest Based on Multi-Stage Knowledge Distillation. Foods.

[5] Lu E., Xu L., Li Y., Tang Z., Ma Z. (2020). Modeling of working environment and coverage path planning method of combine harvesters. Int. J. Agric. Biol. Eng..

[6] Miao Z., Chen S., He C., Jin C., Ma S., Xu S. (2019). Automatic Identification and Location Method of Forage Harvester Trailer Hopper Based on 3D Vision. Trans. Chin. Soc. Agric. Mach..

[7] Happich G., Harms H.-H., Lang T. (2009). Loading of Agricultural Trailers using a Model-Based Method. Agric. Eng. Int. CIGR J..

[8] Liu Z., Jiang C., Evans J.T., Dhamankar S., Heusinger L.J., Shaver G.M., Puryk C.M. (2022). A computationally efficient model for granular material piling in a container. Comput. Electron. Agric..

[9] Dai B.-B., Li T.-Q., Deng L.-J., Yang J., Yuan W.-H. (2022). Fabric effect on the angle of repose in granular materials. Powder Technol..

[10] Wang X., Wang Z., Bai X., Ge Z., Zhao Y. (2022). Dynamic Uniform Loading Method of Grain Box of Transport Vehicle Based on Three-dimensional Point Cloud. Trans. Chin. Soc. Agric. Mach..

[11] Setyawan R.A., Basuki A., Wey C.Y. Machine Vision-Based Urban Farming Growth Monitoring System. Proceedings of the 2020 10th Electrical Power, Electronics, Communications, Controls and Informatics Seminar (EECCIS).

[12] Fonteijn H., Afonso M., Lensink D., Mooij M., Faber N., Vroegop A., Polder G., Wehrens R. (2021). Automatic Phenotyping of Tomatoes in Production Greenhouses Using Robotics and Computer Vision: From Theory to Practice. Agronomy.

[13] Qu H.-R., Su W.-H. (2024). Deep Learning-Based Weed–Crop Recognition for Smart Agricultural Equipment: A Review. Agronomy.

[14] Diao Z., Ma S., Zhang D., Zhang J., Guo P., He Z., Zhao S., Zhang B. (2024). Algorithm for Corn Crop Row Recognition during Different Growth Stages Based on ST-YOLOv8s Network. Agronomy.

[15] Wang H., Lin J., Zou X., Zhang P., Zhou M., Zou W., Tang Y., Luo L. (2024). Construction of a Virtual Interactive System for Orchards Based on Digital Twin. J. Syst. Simul..

[16] Li H., Gu Z., He D., Wang X., Huang J., Mo Y., Li P., Huang Z., Wu F. (2024). A lightweight improved YOLOv5s model and its deployment for detecting pitaya fruits in daytime and nighttime light-supplement environments. Comput. Electron. Agric..

[17] Evarist N., Deborah N., Birungi G., Caroline N.K., Kule B.J.M. (2024). A Model for Detecting the Presence of Pesticide Residues in Edible Parts of Tomatoes, Cabbages, Carrots and Green Pepper Vegetables. Artif. Intell. Appl..

[18] Jahari M., Yamamoto K., Miyamoto M., Kondo N., Ogawa Y., Suzuki T., Habaragamuwa H., Ahmad U. (2015). Double Lighting Machine Vision System to Monitor Harvested Paddy Grain Quality during Head-Feeding Combine Harvester Operation. Machines.

[19] Jiang C., Liu Z., Evans J.T., Shaver G.M., Heusinger L.J., Puryk C.M. (2023). LiDAR-based benchmark approach development and validation for unloading-on-the-go systems incorporating stereo camera-based perception. Biosyst. Eng..

[20] Torii T., Takamizawa A., Okamoto T., Imou K. (2000). Crop row tracking by an autonomous vehicle using machine vision (Part 2)—Field test using an autonomous tractor Piping, Equipment, and Plant Technology. J. Jpn. Soc. Agric. Mach..

[21] Ball D., Upcroft B., Wyeth G., Corke P., English A., Ross P., Patten T., Fitch R., Sukkarieh S., Bate A. (2016). Vision-based Obstacle Detection and Navigation for an Agricultural Robot. J. Field Robot..

[22] Santos L.C., Santos F.N., Valente A., Sobreira H., Sarmento J., Petry M. (2022). Collision Avoidance Considering Iterative Bézier Based Approach for Steep Slope Terrains. IEEE Access.

[23] Bøgelund R.C., Kristian K., Moeslund T.B. (2021). Anchor tuning in Faster R-CNN for measuring corn silage physical characteristics Computers and Electronics in Agriculture. Comput. Electron. Agric..

[24] Zhang W., Gong L., Chen S., Wang W., Miao Z., Liu C. (2021). Autonomous Identification and Positioning of Trucks during Collaborative Forage Harvesting. Sensors.

[25] Ying C., Junmin P. (2002). Volume measurement method of large material stack based on computer vision. J. Shanghai Jiao Tong Univ..

[26] Liu D., Wang Z., Bai X., Zhao Y. Grain Truck Loading Status Detection Based on Machine Vision. Proceedings of the 2019 IEEE 4th International Conference on Image, Vision and Computing (ICIVC).

[27] Cho W., Kurita H., Iida M., Suguri M., Masuda R. (2015). Autonomous positioning of the unloading auger of a combine harvester by a laser sensor and GNSS. Eng. Agric. Environ. Food.

[28] Zhang C., Xu Z., Wang Y., Zhou C. (2015). Study on automatic loading system for bulk grains based on machine vision technology. Hoisting. Convey. Mach..

[29] Cui Z., Hu J., Yu Y., Cao G., Zhang H., Chai X., Chen H., Xu L. (2024). Automatic grain unloading method for track-driven rice combine harvesters based on stereo vision. Comput. Electron. Agric..

[30] Liu Z., Dhamankar S., Evans J.T., Allen C.M., Jiang C., Shaver G.M., Etienne A., Vyn T.J., Puryk C.M., McDonald B.M. (2022). Development and experimental validation of a system for agricultural grain unloading-on-the-go. Comput. Electron. Agric..

[31] Zhang X., Lv T., Dan W., Minghao Z. (2024). High -precision binocular camera calibration method based on a3D calibration object. Appl. Opt..

[32] Zhang H., Lee S. (2024). Advancing the Robotic Vision Revolution: Development and Evaluation of a Bionic Binocular System for Enhanced Robotic Vision. Biomimetics.

[33] Xiaowei S., Gaoyang L., Lei Y., Luxiao Z., Chunping H., Zixiang X. (2022). Real and Pseudo Pedestrian Detection Method with CA-YOLOv5s Based on Stereo Image Fusion. Entropy.

[34] Pal R., Gupta S.K., Ahmad A., Melandsø F., Habib A. (2024). Block-matching and 3D filtering-based denoising of acoustic images obtained through point contact excitation and detection method. Appl. Acoust..

[35] Almatrouk B., Meng H., Swash M.R. (2024). Holoscopic Elemental-Image-Based Disparity Estimation Using Multi-Scale, Multi-Window Semi-Global Block Matching. Appl. Sci..

